# Photo ID verification remains challenging despite years of practice

**DOI:** 10.1186/s41235-018-0110-y

**Published:** 2018-06-27

**Authors:** Megan H. Papesh

**Affiliations:** Department of Psychology, Louisiana State University, Baton Rouge, LA 70803 USA

**Keywords:** Unfamiliar face matching, Expertise, Aging

## Abstract

**Background:**

Matching unfamiliar faces to photographic identification (ID) documents occurs across many domains, including financial transactions (e.g., mortgage documents), controlling the purchase of age-restricted goods (e.g., alcohol sales), and airport security. Laboratory research has repeatedly documented the fallibility of this process in novice observers, but little research has assessed individual differences based on occupational expertise (cf. White et al., PLoS One 9:e103510, 2014; White et al., Proceedings of the Royal Society B 282(1814):20151292, 2015). In the present study, over 800 professional notaries (who routinely verify identity prior to witnessing signatures on legal documents), 70 bank tellers, and 35 undergraduate students completed an online unfamiliar face-matching test. In this test, observers made match/nonmatch decisions to 30 face ID pairs (half of which were matches), with no time constraints and no trial-by-trial feedback.

**Results:**

Results showed that all groups performed similarly, although age was negatively correlated with accuracy. Critically, weekly and yearly experience with unfamiliar face matching did not impact performance.

**Conclusions:**

These results suggest that accumulated occupational experience has no bearing on unfamiliar face ID abilities and that cognitive declines associated with aging also manifest in unfamiliar face matching.

## Significance

The present study examined observers’ ability to match unfamiliar faces to photo identifications (IDs), a task frequently faced by security personnel, clerks, and financial and legal transaction witnesses. This research compared performance of professional notaries public, bank employees, and undergraduates. The results reveal that occupational experience does not improve unfamiliar face-matching ability, and that ability declines with age. These results highlight the need to better understand unfamiliar face identification (ID), and to develop techniques to train individuals responsible for preventing identity fraud.

## Background

In 2008, a group of con artists targeted a relatively wealthy, 75-year-old Palm Springs art dealer named Clifford Lambert (People v. Niroula, Replogle, Garcia, Bustamante, and McCarthy, 2010). After befriending and subsequently murdering Mr. Lambert, two of the co-conspirators requested a notary public to witness a power of attorney transfer, giving one of the accomplices power over an impersonated Mr. Lambert’s bank account. After (wrongly) verifying the signers’ identities, the notary witnessed the transfer and notarized the documents. The criminals then used the power of attorney to obtain approximately US$200,000 from Mr. Lambert’s accounts. Subsequent visits to the notary resulted in powers of attorney transfer for Mr. Lambert’s other assets, including property, accounts, and general estate. Although the notary public failed to spot that the man in front of him did not match the presented ID, he recorded a fingerprint in his notary journal as additional identity verification, which eventually led to the conviction of six co-conspirators. Despite this, the estate of Mr. Lambert was granted a US$10,000 settlement against the notary’s insurance because of the mistaken identity.

Although the Lambert case was exceptional, involving a ring of co-conspirators and murder, identity impersonation can occur in many common financial or property transactions (e.g., divisions of assets, powers of attorney transfers), and it is typically the job of a notary public to ensure that all signers are who they claim to be. Beyond the economic (Harrell & Langton, 2013) and psychological (Golladay & Holtfreter, 2017) consequences for victims, identity fraud can also result in legal action against the notary responsible for verifying identity. For example, notaries can be held civilly and/or criminally liable for failing to properly verify identity (Clarke, 2015). The costs and consequences of identity fraud underscore the need to better understand individual differences unfamiliar face-to-photo matching (i.e., identity verification).

Despite the relative ease with which people recognize highly varied instances of familiar faces (e.g., Burton, Wilson, Cowan, & Bruce, 1999), research consistently shows important differences in the ways that familiar and unfamiliar faces are perceived (Royer et al., 2016; Smith, Volna, & Ewing, 2016) and remembered (Bruce, 1982; Ellis, Shepherd, & Davies, 1979; Hill & Bruce, 1996; Klatzky & Forrest, 1984; O’Toole, Edelman, & Bülthoff, 1998; Patterson & Baddeley, 1977; see Burton, 2013, for a review). Using matching tasks, unfamiliar face perception proves remarkably challenging, both under laboratory (Bruce et al., 1999; Bruce, Henderson, Newman, & Burton, 2001; Megreya & Burton, 2006, 2008) and live (Davis & Valentine, 2009; Kemp, Towell, & Pike, 1997; Megreya & Burton, 2008) conditions. Even under ideal circumstances, such as when both photographs under scrutiny were taken on the same day, performance remains relatively error prone (Bindemann, Avetisyan, & Blackwell, 2010; Megreya & Bindemann, 2015; Megreya, Bindemann, & Havard, 2011). As discussed by Burton (Burton, 2013; also Jenkins, White, Van Montfort, & Burton, 2011), even when two photographs of the same person were taken on the same day, minor variations in pose, lighting, or angles may lead observers to classify the photos as two different people. This seems to reflect within-person variation in photos that exceeds observers’ expectations or tolerance. Indeed, exposing adult participants to variations of the same face produces robust representations of that face, and improved matching performance (Andrews, Jenkins, Cursiter, & Burton, 2015; Baker, Laurence, & Mondloch, 2017; Bindemann & Sandford, 2011; Dowsett, Sandford, & Burton, 2016; Menon, White, & Kemp, 2015a; Ritchie & Burton, 2017).

Although training with multiple images of unfamiliar faces can help observers tolerate within-person variability to extract a constant identity, many occupations require observers to tolerate that variability following exposure to only one photographic identity depiction. For example, security screeners encounter hundreds of people (or more) throughout a given shift, many of whom present identifications containing photos taken years earlier, and under dramatically different conditions (e.g., in lighting, hairstyles, facial hair, skin tones, etc.). Despite such wide variation between people and their ID photos, the vast majority of passengers (presumably) carry valid IDs, so airport screeners must learn to tolerate within-person variability. What are the effects of repeated exposure to such a variable, non-diagnostic signal? Observers’ performance may improve, as experience often improves performance in noisy signal-detection tasks (e.g., medical image scanning; Drew et al., 2013; Kundel & La Follette Jr, 1972; Manning, Ethell, Donovan, & Crawford, 2006; Nodine, Kundel, Lauver, & Toto, 1996). Alternatively, repeated experience with non-diagnostic within-person variability may lead observers to become overly tolerant of variability, missing critical between-individual differences signaling identity imposters (see Papesh & Goldinger, 2014).

To date, relatively few studies have investigated the differences between novices and experts in unfamiliar face matching, and they have yielded mixed results. For example, White, Kemp, Jenkins, Matheson, and Burton (2014) found that Sydney passport officials performed equivalently to undergraduate students, despite having up to 8 years of experience with visual inspection tasks, including unfamiliar face matching. In a subsequent study, however, expertise effects were found in a different population: Forensic facial identification examiners outperformed novices on multiple tests of unfamiliar face perception (White, Phillips, Hahn, Hill, & O’Toole, 2015). Based on larger performance differences observed during longer exposure durations, White et al. suggested that experts adopt a more controlled, feature-by-feature, approach toward the task, consistent with the training that these image analysts receive (Facial Identification Scientific Working Group, 2011).

Although trained forensic examiners outperform novices in tests of unfamiliar face matching, their abilities are called upon only in limited circumstances, and with minimal time pressure. More often, unfamiliar face-to-photo matching occurs under highly variable conditions, including supermarkets, border control and airport security, and in financial transactions. The people making these matching decisions are also highly variable, with individual differences in age, race, cognitive capacities, training, and on-the-job experience. The present research examined individual differences in age and occupational experience, comparing participants who are personally responsible for verifying identity during financial or legal transactions to a control sample of undergraduate students. The primary population of interest included professional notaries in the United States, individuals who are appointed by their state governments to act as impartial witnesses when important financial and legal documents are signed (e.g., property deeds, powers of attorney). In addition to ensuring that proper procedures are followed, including that all signers engage by their own free will, notaries serve a major role in fraud prevention by verifying signers’ identities. The present research addressed several questions about individual differences in unfamiliar face ID by (1) comparing professional notaries and bank tellers (who are also often tasked with unfamiliar face matching) to novices and (2) correlating performance with occupational experience measures, including weekly identity verifications and years of on-the-job experience.

## Methods

### Participants

Thirty-five Louisiana State University students participated for partial course credit (*M*_*age*_ = 21, *SD*_*age*_ = 3.1, 29 women), whereas 67 bank employees (*M*_*age*_ = 38.7, *SD*_*age*_ = 14.6, 58 women) and 1054 notaries public participated voluntarily, for no compensation. Because 206 participants from the notary group did not complete the entire experiment, only 848 notaries (*M*_*age*_ = 50.4, *SD*_*age*_ = 12.8, 728 women) are included in analyses.

### Materials

Stimuli consisted of 30 photograph pairs from the set described by Papesh and Goldinger (2014). The photographs depicted 45 unique identities across 15 matching and 15 mismatching pairs. Within each pair, one face was a standard student ID photo embedded into a mock driver’s license, and the other photo was taken with a digital camera under varied lighting conditions (but with uniform backgrounds). For matching identities, the second photo was taken an average of 1.5 years after the first. Each recent photo was sized to fit an approximate 8″ × 7″-rectangle, and each mock license was sized to fit within a 2.75″ × 4.3″-rectangle. The images were then saved together and scaled to fit within 900 × 700 pixels. No identities appeared in more than one trial.

### Procedure

**Fig. 1:**
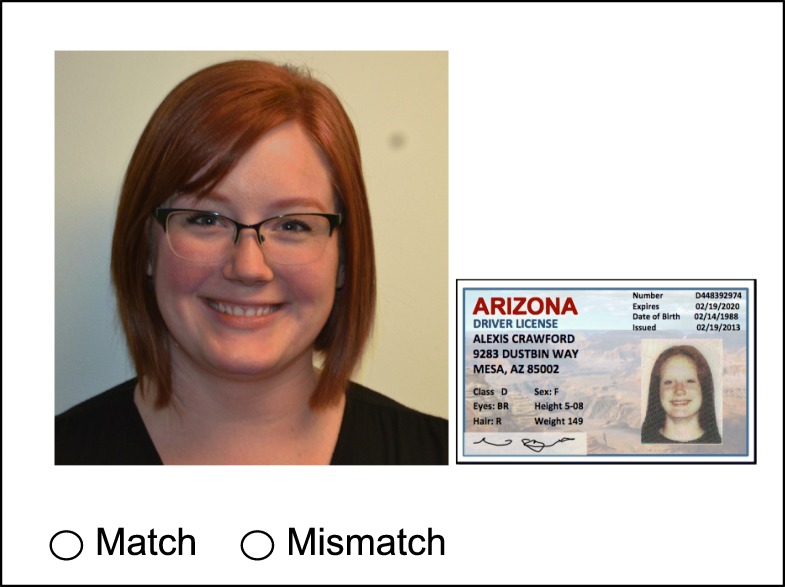
Sample “match” trial. Note that the sample depicts a volunteer who consented to the use of her likeness and was not one of the stimuli used in the experiment

Notaries were recruited via the 2016 and 2017 annual meetings of the National Notary Association (NNA), and from the NNA website. Bank employees were recruited by a bank employee trainer, and undergraduate students were recruited from psychology courses. All participants completed the study online, via Qualtrics. After providing informed consent, participants provided demographic (age, race, sex) and occupational information. Participants employed in occupations requiring unfamiliar face matching also reported the number of years experience in that position and estimated the weekly identity verifications that they performed. Following the demographic survey, participants read instructions explaining that their task was to determine whether two face photographs depicted matching or mismatching identities. They were told that they could ignore the details on the driver’s license,^1^ and to issue their responses via radio buttons appearing below each face (see Fig. 1 for a schematic trial). Matching and mismatching identities appeared in a randomly determined order, one pair at a time, and participants could not skip trials or go back to prior ones. After completing all 30 trials, participants were shown the full stimulus set along with their answers and the correct answers. No trial-by-trial feedback was given (see Alenezi & Bindemann, 2013, for a discussion of the role of feedback in unfamiliar face-matching).

## Results and Discussion

All analyses were performed using both traditional (i.e., frequentist) and Bayesian approaches in JASP (JASP Team, 2018). Because of the inferential power of Bayesian analyses, I report only the outcome of the Bayesian statistics, including Bayes Factors (BFs).^2^ Bayesian priors were set at JASP-recommended defaults, with the *r* scalar for fixed effects at 0.5, random effects at 1.0, and covariates at 0.354 (see Wagenmakers et al., 2018).

**Fig. 2:**
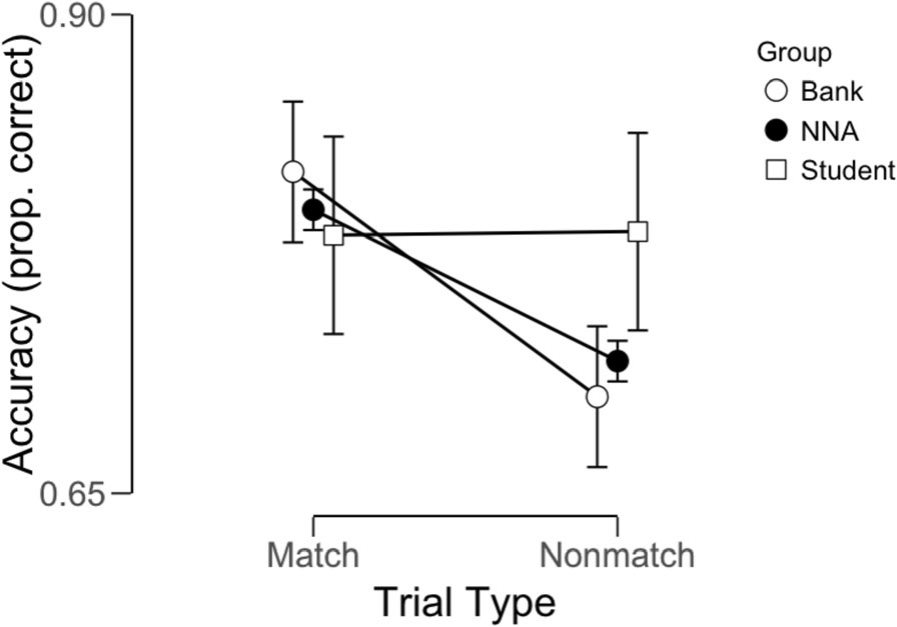
Accuracy on match and nonmatch trials across separate groups. Error bars represent 95% CI

To examine whether professional experience impacts unfamiliar face-matching performance, a 3 (Group: Bank, NNA, Student) × 2 (Trial Type: Match/Mismatch) repeated-measures Bayesian analysis of variance (ANOVA) was conducted, with Age as a covariate. This analysis compared the null model to one involving Age, Group, Trial Type, and the interaction of Group and Trial Type, with *BF*_*Inc*_ representing the strength of evidence favoring the inclusion of each main effect or interaction. The resulting BF_Inc_ were strongly in support of a model including Trial Type (*BF*_*Inc*_ = 6.005e + 15, see Fig. 2) and Age (*BF*_*Inc*_ = 33,513.91), but not Group (*BF*_*Inc*_ = 0.088) or the interaction (*BF*_*Inc*_ = 0.299).

To examine participants’ performance separately from decision biases, I calculated signal detection indexes of *d’* (sensitivity) and *c* (bias). Because of the importance of identifying imposters in applied settings, I treated correct mismatch detections as “hits,” with false positives to matching identities as “false alarms.”^3^ These values were analyzed in separate Bayesian analyses of covariance (ANCOVAs), examining the factor Group, with Age as a covariate. The resulting *BF10* (support for the alternative), *BF01* (support for the null), and *BF*_*Inc*_ are presented in Table 1. As shown, sensitivity was affected by participants’ Age; the directionality of this effect will be discussed in greater detail with subsequent regression and correlational analyses. The Group variable provided greater evidence in favor of the null (BF01), relative to alternative (BF10), revealing that sensitivity did not reliably change across the students (*M* = 1.87, *SD* = .88), NNA members (*M* = 1.70, *SD* = .89), and bank employees (*M* = 1.77, *SD* = .99). Analyses on *c* also revealed a null effect of Group, with similar response biases across the students (*M* = .01, *SD* = .48), NNA members (*M* = .25, *SD* = .54), and bank employees (*M* = .17, *SD* = .51). Unlike *d’* analyses, there was no effect of Age on *c* (see BF01 values, Table 1), suggesting that age-related performance changes are unrelated to participants’ response biases.

**Table 1:** Bayes Factors (BFs) for each predictor variable on *d’* (sensitivity) and *c* (bias)

	*d’*			*c*		
Variable	BF10	BF01	BF_Inc_	BF10	BF01	BF_Inc_
Age	513.66	0.002	503.03	0.09	11.15	0.36
Group	0.08	12.4	0.06	0.36	2.77	0.09

To better understand the influences of age and professional experience on unfamiliar face matching, performance metrics from individuals with occupational face-matching experience^4^ were analyzed in separate Bayesian linear regressions, with Age, Weekly Experience, and Years of Experience as predictors. As shown in Table 2, Age clearly influenced accuracy measures (match accuracy, mismatch accuracy, and *d’*) without affecting criterion (*c*). The reliable effects are considered “extremely strong” for matches and *d’*, and “very strong” for mismatches. The evidence for the experience variables, however, was clearly in favor of the null model (BF01 column) for all performance measures, with evidence ranging from “moderate” to “strong.”

**Table 2:** Bayes Factors (BFs) for each predictor variable on accuracy and signal-detection measures

	Match accuracy			Mismatch accuracy			*d’*			*c*		
Variable	BF10	BF01	BF_Inc_	BF10	BF01	BF_Inc_	BF10	BF01	BF_Inc_	BF10	BF01	BF_Inc_
Age	123.22	0.008	138.92	40.14	0.03	62.66	594.26	0.002	775.13	0.08	13.38	0.09
Weekly experience	0.02	5.09	0.13	0.11	8.94	0.63	0.09	10.86	0.13	0.35	2.85	0.09
Years of experience	0.08	12.11	0.31	0.09	10.13	0.19	0.08	11.89	0.39	0.08	12.87	0.38

**Fig. 3:**
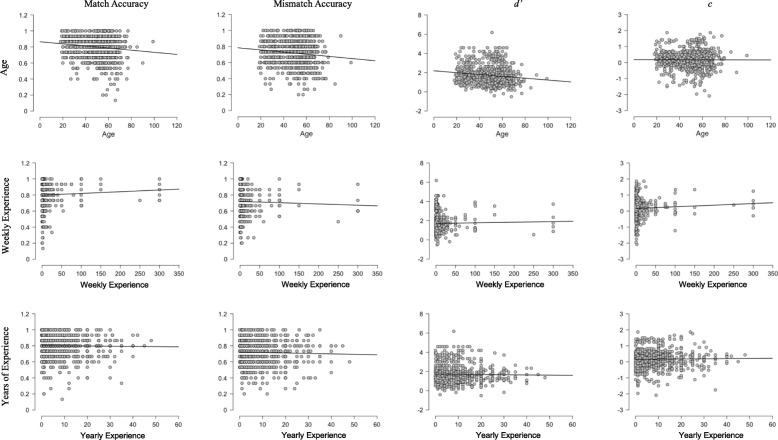
Scatterplots of the correlations between performance metrics (identified by the columns) and individual differences measures (identified by the rows). Corresponding sequential analyses showing the strength of evidence can be found in the Appendix

Because the overwhelming evidence from the regression analyses was in favor of the null hypothesis (with the exception of the influence of Age), I examined Bayesian correlation pairs between each predictor and outcome variable. With Bayesian correlation pairs, JASP provides both correlation plots and sequential analyses, showing the accumulation of evidence favoring (or not favoring) a correlation between the two variables. This information is useful for understanding the impact of the variable, and the role of sample size in determining evidence. The hypothesis under test was that the variables were correlated, without any directional assumptions. The outcome of these correlations is shown in Fig. 3.

As shown in Fig. 3 (and confirmed by the sequential analyses in the Appendix), the strength of evidence favoring a correlation between age and accuracy ranged from strong (mismatching identities) to extreme (matching identities, *d*’). Although there was a positive correlation (*r* = .45, *CI*[.39, .50]) between age and years of experience, the correlation between age and both performance measures was negative (Match *r* = − 13, Mismatch *r* = − 12). All of the correlations between experience measures and outcome measures favored the null hypothesis (see column 4 and the middle and bottom rows of the sequential analyses), with moderate to strong evidence, suggesting that there is no influence of weekly ID verifications or years of experience on unfamiliar face-matching ability.

**Fig. 4:**
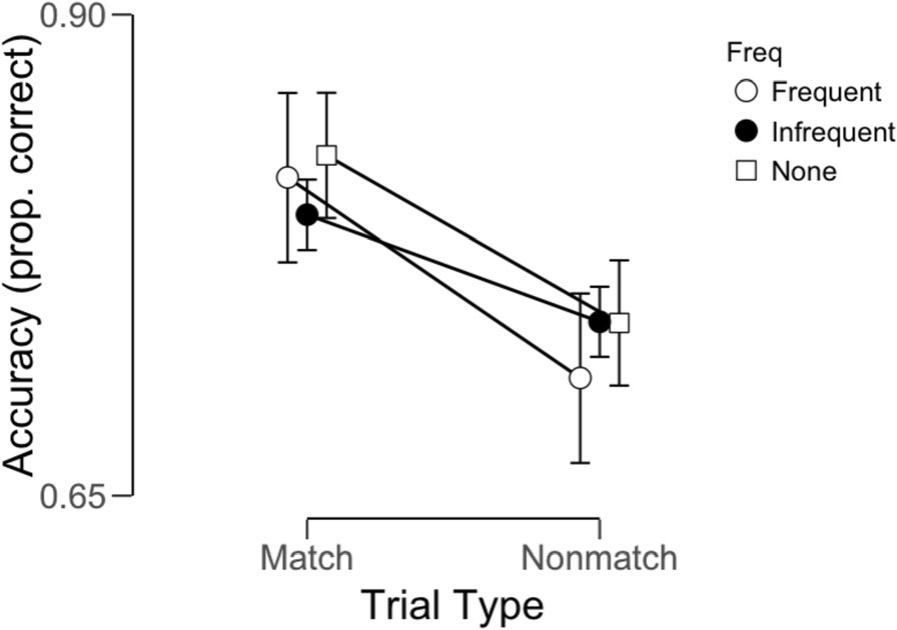
Match and nonmatch accuracy for age-matched participants with varied weekly ID verification frequency. Error bars represent 95% CI

Lastly, to determine whether professional experience impacts performance when age is held constant, I selected 30–50-year-old professionals, and classified their weekly ID matching experience as “never” (zero IDs in any given week^5^; *n* = 62, *M*_*age*_ = 39.4 years), “infrequent” (fewer than 10 IDs checked in an average week; *n* = 217, *M*_*age*_ = 41.7 years), or “frequent” (more than 10 per average week; *n* = 39, *M*_*age*_ = 40.8 years). Importantly, this subset of participants was younger than 65 years, which has previously been identified as a cohort with age-related declines in face matching (Megreya & Bindemann, 2015). A 2 (Trial Type: match/nonmatch) × 3 (Frequency) Bayesian ANOVA revealed a strong effect of Trial Type (BF10 = 1.913e + 8; *BF*_*inc*_ = 1.292e + 8) and strong null effects (BF01 values > 18) of every other effect and interaction. As shown in Fig. 4, participants were more accurate on match, relative to nonmatch, trials, and this effect was consistent across levels of professional experience. Follow-up analyses on *d’* and *c* revealed clear null effects of Frequency (*d’* BF01 = 10.78; *c* BF01 = 5.09), further confirming that individual differences in professional experience do not mediate unfamiliar face matching.

## Conclusions

The present study compared three groups of individuals with varied occupational experience in unfamiliar face matching. Notaries, who are often legally required to hold liability insurance to protect them in the event of bad notarizations (including those resulting from identity impersonation), bank tellers, who are professionally obligated to verify identity for financial transactions, and undergraduate students completed a brief online unfamiliar face-matching test. Despite their years of experience and weekly identity verifications, both professional groups performed similarly to the student group. Indeed, the only reliable individual difference predictor of performance was age, such that increasing age predicted declining performance. Unlike the forensic image experts studied by White et al. (2015), however, the professionals in the present study received no formal image comparison training, and were only “trained” via on-the-job experience.

Beyond the effect of age, the only other reliable effect was that of trial type (i.e., whether observers were viewing matching or nonmatching identities). The majority of participants were more accurate when the to-be-matched identities depicted the same person, relative to two different people. Others have observed similar effects (e.g., Bindemann et al., 2010; Bindemann, Avetisyan, & Rakow, 2012; Bobak, Dowsett, & Bate, 2016; Kokje, Bindemann, & Megreya, 2018), although not always (e.g., Fysh & Bindemann, 2017). Moreover, some have suggested that matching and mismatching decisions (or “target-present” and “target-absent” decisions in lineup studies) reflect dissociable processes, such that the ability spot identity matches is unrelated to the ability to reject mismatching identities (Bruce et al., 1999; Burton, White, & McNeill, 2010; Megreya & Burton, 2007). In the present study, participants were clearly better able to correctly match identities, relative to identifying mismatching identities. The underlying processes responsible for this difference remains an open question for future research.

Although age and years of occupational experience were (obviously) positively correlated, age was negatively correlated with unfamiliar face-matching performance, and occupational experience factors had no impact on ability, even when age was held constant. Prior work investigating unfamiliar face-matching and developmental prosopagnosia observed a similar, but small, decline in performance with age (Shah, Sowden, Gaule, Catmur, & Bird, 2015). The present study provides evidence that the impact of age on unfamiliar face matching is strong and outweighs the influence of relevant occupational experience. The overwhelming effect of age could have multiple causes (see Megreya & Bindemann, 2015). For example, older adults have more experience tolerating within-person variability in their friends and family, which may lead them to become overly tolerant of similar variability in unfamiliar faces. Alternatively, the cognitive decline commonly associated with non-pathological aging (e.g., McNab et al., 2015), or reduced neural specialization in the ventral visual cortex, which is often recruited for face-specific processing (Park et al., 2004), may lead to performance declines. The latter explanation becomes less tenable in light of studies showing spared face-specific processing in older adults (e.g., Boutet & Faubert, 2006; Konar, Bennett, & Sekuler, 2013; Meinhardt-Injac, Persike, & Meinhardt, 2014), and is not considered further.

The influence of variability on unfamiliar face perception has become better appreciated in recent years (see Burton, 2013), and is critical for the development of robust face representations. For example, Baker et al. (2017) found that, when participants experienced perceptual variability in depictions of a face, they developed more stable face representations for that face, allowing them to appreciate different photos of the same person as representing the same person (see also Menon, White, & Kemp, 2015b; Ritchie & Burton, 2017). Although exposure to within-person variability may aide face *recognition*, it is perhaps detrimental to face matching, as observers learn to become overly tolerant of wide variability. As noted by Jenkins et al. (2011), myriad facial changes occur across days, weeks, months, and years. By repeatedly experiencing and discounting these changes, older adults may become complacent with regard to featural variability diagnostic of identity impersonation. Although this may explain the impact of age on mismatching identity performance, it does not address why age was also negatively correlated with performance on matching identities.

Perhaps the best (current) explanation for the powerful effect of age on unfamiliar face matching performance is the well-established decline of cognitive capacities served by the frontal lobes, such as working memory capacity (WMC, McNab et al., 2015). With healthy aging, older adults compensate for reductions in WMC, particularly inhibitory processing, with greater focused attention. During demanding perceptual tasks, older adults may engage greater attentional resources, leaving fewer available for the other cognitive operations. In face-matching, these remaining cognitive operations may involve both perceptual and decisional processes. For example, Megreya and Bindemann (2015) found age-related declines in face matching and suggested that the detrimental effects were due to declines in older adults’ ability to perceptually encode unfamiliar faces. The present results are consistent with this finding, and the signal detection analyses showed clear negative effects of age on *d’* (sensitivity), but not *c* (criterion). This suggests that aging selectively impairs the perceptual processes involved in unfamiliar face matching, leaving the criterion-setting aspect of decision-making unaffected.

Although the influence of age on unfamiliar face matching is theoretically and practically interesting, the more compelling individual differences in the present study were related to the effects of weekly experience and years of professional, on-the-job, experience. These experience variables yielded null effects in traditional (frequentist) testing, which would be difficult to interpret without Bayesian analyses. With Bayesian analyses, however, researchers can properly test the null hypothesis, and overcome publication bias against null results, which can impede scientific progress (Ferguson & Heene, 2012). In the present research, the null hypothesis was that weekly and yearly experience do not impact observers’ ability to verify identity matches or spot mismatches. This counterintuitive hypothesis was moderately to strongly supported by both the Bayesian ANOVA and linear regressions.

Unlike other visual scanning domains (e.g., medical image screening, baggage screening, forensic image analysis), the professionals in the current study received no specific, focused training in their skill. Relative to novices, experts in visual search have improved scanning time/efficiency (Biggs, Cain, Clark, Darling, & Mitroff, 2013; Biggs & Mitroff, 2014; Kundel & La Follette Jr, 1972; Manning et al., 2006; Nodine et al., 1996; Spitz, Put, Wagemans, Williams, & Helsen, 2016; Wood, 1999), enhanced detection accuracy (Biggs & Mitroff, 2015; White et al., 2015), and extract more information from visual displays (Charness, Reingold, Pomplun, & Stampe, 2001; Reingold & Charness, 2005; Reingold, Charness, Pomplun, & Stampe, 2001). In the present study, the professionals showed no evidence of improved detection accuracy, which was the primary focus of the investigation. Instead, their performance strongly suggested that accumulated experience had no impact on their ability to perform the task. To improve performance, and reduce the likelihood of identity imposters’ success, professionals tasked with verifying identity should perhaps be trained in a manner similar to forensic image analysts, who are the only documented professional group to outperform novices on unfamiliar face matching (White et al., 2015; see Bobak et al., 2016 and Russell, Duchaine, & Nakayama, 2009, for discussion of potential “super recognizers”).

In addition to revealing the detrimental role of aging in unfamiliar face matching, the present results add to the growing academic literature examining the impact of training and occupational experience on face-matching performance. Consistent with the passport officers studied by White et al. (2014), professionals in the present study performed similarly to undergraduate students. This suggests that laboratory tests of unfamiliar face matching using convenience samples of college students are good approximations for other populations, and it also suggests that passive occupational experience does not benefit observers. Instead, the only occupational experience currently known to improve face-matching abilities involves dedicated training (White et al., 2015) and contextual circumstances without time pressure. Together, these findings implicate specific training as one method to potentially improve performance, and potentially reduce the personal and economic impact of identity fraud.

## Figures and Tables

**Fig. 5:**
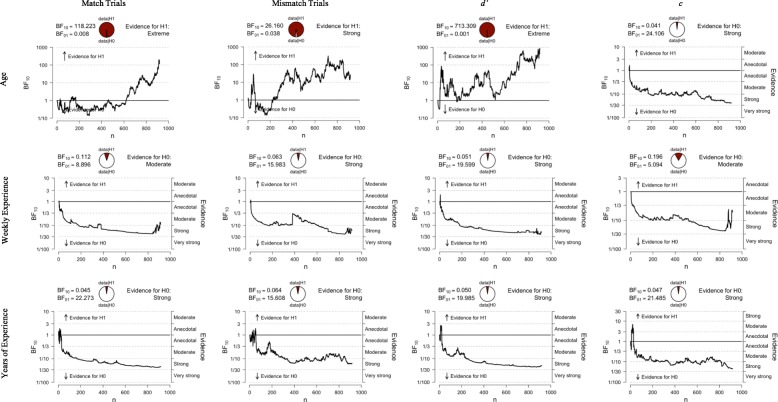
Sequential analyses showing the influence of age (top row), weekly experience (middle row), and years of experience (bottom row) on match (left column) and mismatch (right column) accuracy. Resulting BF10 values are plotted as additional participants are added to the analysis, with evidence labels on the right of the graphs showing evidence favoring the null hypothesis.

## Abbreviations

ANOVA: Analysis of variance
BF: Bayes Factor
ID: Identification
WMC: Working memory capacity

## Footnotes

1 McCaffery and Burton (2016) found that biographical details impair mismatch detection. Although this may be true in the present study as well, the presence of mock biographical details renders the task a more realistic approximation of applied face-matching.

2 Full frequentist and Bayesian analyses can be found here, along with the entire dataset: https://osf.io/mr3sq/?view_only=fda710c37a2644b98c0bb0aa39575eda.

3 Mathematically, *d’* was unaffected when correct and incorrect “match” decisions formed the basis of the hit and false alarm rates, respectively.

4 This included all of the Notary and Bank participants, but only 10 Student participants

5 These individuals were mostly notaries who had recently been licensed, but not yet started practicing.
